# Effectiveness of Interventions on Psychological Resilience among Individuals with Cancer: A Systematic Review and Meta-analysis

**DOI:** 10.1192/j.eurpsy.2022.228

**Published:** 2022-09-01

**Authors:** Y.S. Üzar-Özçetin, S. Öcalan

**Affiliations:** Hacettepe University, Psychiatric Nursing, Ankara, Turkey

**Keywords:** cancer, patient, resilience, meta-analysis

## Abstract

**Introduction:**

Cancer is a disease that can cause traumatic experiences and disrupt the balance of life in individuals. The development of resilience in individuals is important in adapting to the cancer process and the difficulties that the process may bring.

**Objectives:**

To investigate the effectiveness of interventions for the psychological resilience of individuals with cancer.

**Methods:**

The findings of randomized controlled trials related to interventions to effect resilience of individuals with cancer were included. Comprehensive Meta-Analysis software was used to analyze the data. Hedges’ g and 95% confidence intervals were computed to estimate the effect. Additionally, funnel plots were created to assess publication bias. The Preferred Reporting Items for Systematic Reviews and Meta‐Analysis was used.

**Results:**

The eight studies that demonstrated the effect of the interventions on resilience of individuals with cancer were heterogeneous. The effect size was 2.649 (95% CI = 1.325 - 3.973), was statistically significant (p < 0.001). Results of the subgroup analysis showed that the effects of sample size, cancer type, lenght of treatment, duration of intervention and gender were significant. According to the lenght of treatment studies lasting >90 min and >10 weeks were less significant impact on resilience. Studies in which the sample consisted of more than 100 participants, conducted participants with mixed type of cancer and the sample consisted of participants from both genders demonstrated statistically significant effects on resilience.

**Conclusions:**

This study showed that supportive interventions are crucial in developping psychological resilience among individuals with cancer. However, the findings also underscore the need for further research.

**Disclosure:**

No significant relationships.

